# Editorial: Lifestyle Psychiatry

**DOI:** 10.3389/fpsyt.2019.00597

**Published:** 2019-08-26

**Authors:** Joseph Firth, Philip B. Ward, Brendon Stubbs

**Affiliations:** ^1^NICM Health Research Institute, Western Sydney University, Westmead, NSW, Australia; ^2^Division of Psychology and Mental Health, Faculty of Biology, Medicine and Health, University of Manchester, Manchester, United Kingdom; ^3^Centre for Youth Mental Health, University of Melbourne, Melbourne, VIC, Australia; ^4^School of Psychiatry, UNSW Sydney, Sydney, NSW, Australia; ^5^Schizophrenia Research Unit, Ingham Institute of Applied Medical Research, Liverpool, NSW, Australia; ^6^Physiotherapy Department, South London and Maudsley NHS Foundation Trust, London, United Kingdom; ^7^Department of Psychological Medicine, Institute of Psychiatry, Psychology and Neuroscience, King’s College London, London, United Kingdom

**Keywords:** physical activity, exercise, nutrition, diet, sleep

## What Is Lifestyle Psychiatry?

As the interface between physical and mental health becomes more widely acknowledged (1), the role of “lifestyle factors” (i.e., health behaviors, such as physical activity, diet, and sleep) in the onset and treatment of psychiatric disorders is also gaining increasing interest. Within this Research Topic on “Lifestyle Psychiatry” (a term popularized by Noordsy) (2), we set out to examine how multiple different aspects of our lifestyles relate to our mental health. More specifically, the articles in this collection set out to further articulate how symptoms of mental illness are related to typical health behaviors (including exercise, diet, and sleep) while also presenting new considerations for “lifestyle psychiatry,” such as mindfulness, stress management techniques, and digital technology use. The main findings in each section are presented below.

### Traditional Health Behaviors and Lifestyle Psychiatry

Physical activity, nutrition, and sleep are all widely regarded as fundamental aspects of human health, for both the body and the mind. The role of physical activity in psychiatry is particularly well researched, with a number of recent meta-analyses showing that physical activity can aid in the prevention (3, 4) and management (5–9) of multiple symptoms of mental illness. Indeed, recent international guidelines now recommend exercise as a first-line treatment for mild/moderate depression and as an adjunctive intervention for severe mental illness (10). Building on this evidence, Hegberg et al. produce a new review on how exercise may also be helpful for those with PTSD. By drawing on data from 19 different studies, they explore the efficacy and underlying mechanisms of exercise as an intervention PTSD and present rationale for implementation. In further examination of the physical–mental health interface, Roeh et al. systematically review the evidence for using exercise to reduce symptoms of depression in those with *physical conditions* (rather than solely mental illness). Of the 39 meta-analyses examining this broad topic, 33 found positive effects of exercise on depressive symptoms as a comorbidity to cancers, cardiovascular diseases, and neurological conditions Roeh et al. Although more rigorous large-scale studies are needed, the existing evidence base shows that exercise is not only beneficial in the treatment of mental illnesses but can also be helpful for those experiencing subthreshold symptoms of mental illness as a comorbidity to physical illness.

Given the now substantial evidence base for exercise and mental health, the key outstanding question now becomes about motivating among individuals with mental illness to engage in sufficient exercise in order to feel these beneficial effects. Ho et al. shine new light on this with regards to severe mental illness, in a mixed-methods study of individuals with schizophrenia’s experiences and reasons for exercise. Interestingly, participants with schizophrenia emphasized their core reasons for exercising were largely for the short-term improvements in mood, overall feelings of well-being, and boosting cognition (rather than for psychotic symptoms). The importance of understanding the motivations for exercise among those with mental illness is further emphasized by two other studies in this Research Topic, by Scheewe et al. and Engh et al. Together, these studies show how people with schizophrenia who engage in higher levels of physical activity have greater cardiorespiratory fitness, which is in turn associated with better physical and mental health outcomes of this condition.

Much like physical activity, our dietary food intake is a core determinant of physical health. Now, the potential impact of nutrition on mental health is also gaining increasing recognition due to large-scale meta-analyses of randomized controlled trials (RCTs) showing that both dietary interventions (11) and certain nutrient supplements (12) can significantly reduce symptoms of various psychiatric disorders. Adding to the evidence in this Research Topic, Marotta et al. present findings from a new double-blind, placebo-controlled RCT of probiotic supplementation. The findings confirm a beneficial effect of probiotics for self-reported mood and sleep quality after just 6 weeks, although further research is required to determine if these benefits extend to populations with clinically diagnosed psychiatric disorders.

In a longitudinal investigation of over 3,000 adolescents, Hoare et al. presents equivocal data on the link between nutrition and mental health, showing that the association between fruit/vegetable consumption in adolescents and reduced risk of depression is nonsignificant after adjusting for confounding factors. However, the relationship between healthy diet and healthy mind is unlikely to persist when examining only specific food groups—and may be better conceptualized through a “whole of diet” approach. Specifically, the overall “inflammatory potential” of the diet can now be calculated through scoring an individual’s relative intake of “anti-inflammatory” nutrients (such as fruits, vegetables, and whole foods) against their intake of “inflammatory” foods (typically from calorie-dense processed foods and refined carbohydrates). Owing to the link between heightened inflammatory status and various mental disorders (including depression, bipolar disorder, and schizophrenia), the inflammatory potential of the diet is one key pathway thought to underlie associations between nutrition and mental health (13–15). A cross-sectional study by Shivappa et al. supports this link, showing that female adolescents with the most proinflammatory diets have around four times higher odds for moderate depressive symptoms compared to female adolescents with least inflammatory diets.

Despite these strong cross-sectional associations, there is now an urgent need to move beyond observational evidence of the link between nutrition and mental health, as such studies fail to tease out the bidirectional nature of poor diet and mental illness. Indeed, in this same Research Topic, Teasdale et al. examine the link from the opposite direction, presenting compelling new data to show that the poor diet and excessive energy intakes, which has previously been observed in those with long-term schizophrenia (13, 16), is also observed in young people undergoing initial treatment for this condition. Of note, Teasdale et al. discuss how this is a consequence (rather than cause) of their condition, due to the appetite-inducing effects of antipsychotic medications.

Similarly, sleep disturbance also holds bidirectional associations with mental illness, occurring both as a causal factor and consequence of mental ill-health (8, 17), which also interacts with other lifestyle factors. A first-in-field study by Waite et al. sheds new light on the reciprocal relations between sleep and psychological functioning in young people with indicated mental illness, using rigorous qualitative methods to detail both the impact of sleep disturbance, and need for sleep interventions, in high risk groups. Crucially, two further studies in this Research Topic confirm that exercise, diet, and sleep can all be addressed concurrently, with pilot data from Deenik et al. and Murphy et al. demonstrating the feasibility, along with potential physical and mental health benefits, of lifestyle interventions which combine exercise, diet, and sleep components into a single intervention for people with severe mental illness.

### Emerging Considerations in Lifestyle Psychiatry

In the fast-growing field of Lifestyle Psychiatry, novel interventional approaches are emerging as potential self-management techniques for mental health and well-being. One such example is “mindfulness.” In this Research Topic, Lu et al. found that mindfulness behaviors play a central role in alleviating the adverse mental health outcomes of stressful working conditions in intensive care nurses. Alongside mindfulness, Sarris et al. discusses how other interventions such as yoga, breathing techniques, nature therapies, and light, heat, and art-based therapies could be considered within the broader framework of Lifestyle Psychiatry. However, further RCTs are still required to establish if such interventions are only useful for promoting mental well-being in generally healthy samples or if these approaches can actually provide effective adjunctive treatment for those with severe mental illness.

Additionally, a “newer” aspect of our lifestyle which is currently overlooked in psychiatry is our engagement with digital technologies—particularly smartphones and connected devices. Whereas a recent major review has identified several pathways through which digital technology use can influence cognition (18), the role of these devices in mental healthcare is not well characterized. Nonetheless, the study by Hoffman et al. provide new insights on the applicability of these technologies for delivering behavioral health services, showing high acceptability and broad endorsement (among both patients and providers) for using digital devices for delivering mental health care through primary care settings. Considered alongside the indicated efficacy of digital interventions for both common and severe mental disorders (14–21), now is clearly the time for more rigorous investigation into how these new tools can be integrated within existing care systems.

Along with providing new interventions options for “Lifestyle Psychology,” digital technologies can also facilitate the gathering of lifestyle data. Indeed, papers by Berry et al. and Faulkner and Sidey–Gibbons, in this Research Topic highlight the inadequacy of existing measures, and Shoval et al., indicates a need for more fine-grain analysis of the relationship between physical and mental health status in people with mental illness. Capitalizing on the opportunities afforded by digital devices for passive and active data collection presents a promising path forward for Lifestyle Psychiatry in order to capture the level of detailed data necessary for better characterizing the dynamic relations between physical health behaviors and mental health symptoms.

### Next Steps for Lifestyle Psychiatry

Overall, the studies within this Research Topic covers a broad range of health behaviors, which may play a central role in the prevention and self-management of mental illness. Along with the established fundamentals of human health (such as diet, exercise, and sleep), several other factors (such as stress management, nature time, and digital technology usage) should also be considered within Lifestyle Psychiatry, as this field continues to progress. The priorities for future research now lay with regards to i) moving from the wealth of observational studies to now focus on examining both the efficacy and implementation of lifestyle-focused approaches in mental healthcare, ii) harnessing digital technologies to facilitate the assessment of physical–mental health interactions, and delivery of lifestyle psychiatry interventions, and (iii) considering lifestyle factors in combination, rather than individual behaviors in isolation—in order to develop a more complete understanding of the interactions between our lifestyle and mental well-being. Ultimately, rigorous further research on the use and implementation of multicomponent lifestyle interventions could not only improve our understanding of the interface between physical and mental health but also align these existing individual fields of research (on physical activity, diet, etc.), to present more comprehensive and effective lifestyle treatment programs for individuals with mental illness.

## Author Contributions

All authors contributed equally to the writing of this manuscript, and have approved the final version.

## Funding

JF is supported by a Blackmores Institute Fellowship. JF declares that as a medical research institute, NICM Health Research Institute receives research grants and donations from foundations, universities, government agencies, and industry. Sponsors and donors provide untied and tied funding for work to advance the vision and mission of the Institute. BS is supported by a Clinical Lectureship (ICA-CL-2017-03-001) jointly funded by Health Education England (HEE) and the National Institute for Health Research (NIHR). BS is part funded by the NIHR Biomedical Research Centre at South London and Maudsley NHS Foundation Trust. BS is also supported by the Maudsley Charity, King’s College London and the NIHR South London Collaboration for Leadership in Applied Health Research and Care (CLAHRC) funding. This paper presents independent research. The views expressed in this publication are those of the authors and not necessarily those of the acknowledged institutions.

## Conflict of Interest Statement

The authors declare that the research was conducted in the absence of any commercial or financial relationships that could be construed as a potential conflict of interest.
